# Supplementary material for: Effect of emotional factors on purchase intention in live streaming marketing of agricultural products: A moderated mediation model
Source: PLoS One. 2024 Apr 1;19(4):e0298388. doi: 10.1371/journal.pone.0298388 (PMC10984517; doi:10.1371/journal.pone.0298388)
Supplement: S1 File — (DOCX) [file pone.0298388.s001.docx]

**农产品直播中情感因素影响研究调查问卷**

尊敬的女士/先生：

感谢您愿意在百忙之中填写此份问卷。我们是来自北华航天工业学院的研究团队，正在进行一项农产品直播中情感因素影响作用的研究，需要收集相关数据。本调查采用无记名形式，不采集您的个人敏感信息，您所填的内容仅供调查研究之用，我们会对采集的数据脱敏处理并严格保密、妥善保存，保证不会对您造成任何不良影响。我们希望您能够根据自己的真实看法来回答相关问题。感谢您支持我们的工作！如果您尚未年满16周岁，请直接退出调查。

一、基本情况

1.您的性别：

○男

○女

2.您的年龄：

○20岁及以下

○21-30岁

○31-40岁

○41-50岁

○51岁及以上

3.您近半年的常住地为：

○城市

○乡村

4.您的学历：

○初中及以下

○高中/中专

○本科/专科

○研究生

5.您的职业：

○学生

○党政机关/事业单位工作人员

○企业员工

○自由职业者

○全职妈妈/爸爸

○退休人员

○其他

6.您每月的可支配收入大约为:

○1000元以下

○[1000元，4000元)

○[4000元，7000元)

○[7000元，10000元)

○10000元及以上

7.您是否观看过农产品电商直播：

○是

○否 (退出调查)

8.您最近两周内是否较为完整地观看过农产品直播（观看时长不低于20分钟，主播完整介绍完某一款产品），接下来的问题需要您回忆近两周内观看过的令您印象最深刻的一次直播，并以此次直播为对象，完成后面几个问题的回答。

○是

○否 (退出调查)

1. 下列各题旨在了解您对农产品直播营销的相关态度，请依据实际情况在适当的数字上打√。1=非常不同意；2=不同意；3=有点不同意，4=没意见；5=有点同意；6=同意；7=非常同意。

9.您对三农的看法。

| 我认为悠久灿烂的中华农耕文化需要传承下去。 | 1 | 2 | 3 | 4 | 5 | 6 | 7 |
| --- | --- | --- | --- | --- | --- | --- | --- |
| 民以食为天。我认为发展好农业是安天下、稳民心的战略需要。 | 1 | 2 | 3 | 4 | 5 | 6 | 7 |
| 我认为让农民过上好日子是一件很重要、值得去做的事。 | 1 | 2 | 3 | 4 | 5 | 6 | 7 |
| 我有一个诗画田园的梦想。 | 1 | 2 | 3 | 4 | 5 | 6 | 7 |

10.您观看农产品直播的情况。请以第8题您回想的那次直播为对象，完成本题的回答。

| 该直播间对农产品的包装、食用方法等内容进行了详细和直观的展示。 | 1 | 2 | 3 | 4 | 5 | 6 | 7 |
| --- | --- | --- | --- | --- | --- | --- | --- |
| 该直播间的主播能够与消费者亲切交流，及时、耐心地解答消费者的疑问。 | 1 | 2 | 3 | 4 | 5 | 6 | 7 |
| 该直播间安排了丰富的互动活动（如点赞、参与截屏抽奖、抢红包等）。 | 1 | 2 | 3 | 4 | 5 | 6 | 7 |
| 在观看该直播间的农产品直播时，我可以与其他消费者分享交流农产品相关信息、讨论直播内容。 | 1 | 2 | 3 | 4 | 5 | 6 | 7 |
| 当我观看直播时，该直播间的直播场景（果园、农田、市场、加工厂等）我非常熟悉。 | 1 | 2 | 3 | 4 | 5 | 6 | 7 |
| 该直播间的直播场景让我有一种身临其境的感觉。 | 1 | 2 | 3 | 4 | 5 | 6 | 7 |
| 该直播间主播对上架农产品相关内容的讲解，画面感很强。 | 1 | 2 | 3 | 4 | 5 | 6 | 7 |
| 在观看该农产品直播时，我感觉直播中的农产品就在我眼前，触手可及一般。 | 1 | 2 | 3 | 4 | 5 | 6 | 7 |
| 我相信该直播间上架农产品的相关信息真实可靠。 | 1 | 2 | 3 | 4 | 5 | 6 | 7 |
| 我相信该直播间提供的产品和服务是有质量保证的。 | 1 | 2 | 3 | 4 | 5 | 6 | 7 |
| 我相信我下单后收到的产品与直播间的描述一致。 | 1 | 2 | 3 | 4 | 5 | 6 | 7 |
| 我相信从该直播间所购产品的品质与我的预期一致。 | 1 | 2 | 3 | 4 | 5 | 6 | 7 |
| 我相信该直播间能够积极履行对消费者所作的承诺。 | 1 | 2 | 3 | 4 | 5 | 6 | 7 |
| 在该直播间里听主播讲述农村生活和农业生产场景时，我感觉这些场景、这些人和事就发生在我身边。 | 1 | 2 | 3 | 4 | 5 | 6 | 7 |
| 在该直播间里听主播讲述农村生活和农业生产场景时，我感觉自己就是其中一员。 | 1 | 2 | 3 | 4 | 5 | 6 | 7 |
| 我觉得主播讲述的农产品背后的故事很有趣，我听得很投入、很感动。 | 1 | 2 | 3 | 4 | 5 | 6 | 7 |
| 在该直播间观看农产品直播时，我就像在跟老朋友聊家常、聊收成。 | 1 | 2 | 3 | 4 | 5 | 6 | 7 |
| 在观看该农产品直播过程中，我第一时间就成功下单。 | 1 | 2 | 3 | 4 | 5 | 6 | 7 |
| 在观看该农产品直播时，我将上架产品加入了购物车，方便日后比较并购买。 | 1 | 2 | 3 | 4 | 5 | 6 | 7 |
| 我愿意第一时间把该直播链接转发给好友。 | 1 | 2 | 3 | 4 | 5 | 6 | 7 |
| 当主播或农户在直播间提到农产品滞销困境时，我愿意下单支持农户。 | 1 | 2 | 3 | 4 | 5 | 6 | 7 |

调查到此结束，再次感谢您的支持和帮助！
