# Supplementary material for: Effect of emotional factors on purchase intention in live streaming marketing of agricultural products: A moderated mediation model
Source: PLoS One. 2024 Apr 1;19(4):e0298388. doi: 10.1371/journal.pone.0298388 (PMC10984517; doi:10.1371/journal.pone.0298388)
Supplement: S2 File — (DOCX) [file pone.0298388.s002.docx]

**Questionnaire about effect of emotional factors on purchase intention in streaming live marketing of agricultural products**

Dear lady/sir:

Thank you for filling out this questionnaire in your busy schedule. We are a research team from the North China Institute of Aerospace Technology, and we are conducting a study on the effect of emotional factors on purchase intention in streaming live marketing of agricultural product. We need to collect relevant data. This questionnaire are anonymous, and your personal sensitive information will not be collected. The answers you fill in are only for statistical research. We will desensitize the collected data and keep it strictly confidential and properly stored to ensure that it will not have any adverse effects on you. We sincerely hope you can answer relevant questions according to your true views. If you are not yet 16 years old, please withdraw from the survey directly. Thank you for your help!

***A. Interviewee’s Basic Information***

1. Your gender:

○Male

○Female

1. Your age:

○Under 20 years

○21–30 years

○31–40 years

○41–50 years

○51 years and older

1. Your residence in the past six months:

○Urban area

○Countryside

1. Your education background:

○Junior high school and below

○High school/technical school

○Undergraduate/junior college

○Postgraduate

1. Your Occupation:

○Student

○Government agencies/public institutions

○Enterprise employees

○Freelancer

○Stay-at-home parents

○Retired

○Others

1. Your monthly disposable income:

○1,000 yuan RMB and below

○[1,000 yuan RMB, 4,000 yuan RMB)

○[4,000 yuan RMB, 7,000 yuan RMB)

○[7,000 yuan RMB, 10,000 yuan RMB)

○Above 10,000 yuan RMB

7. Have you watched streaming live marketing of agricultural product?

○ Yes

○ No (exit this questionnaire)

8.Have you watched a complete live streaming of agricultural products in the past two weeks？(The viewing time shall not be less than 20 minutes, and the hosts shall fully introduce a certain product. The following questions require you to recall the most impressive live streaming you watched in the past two weeks, and complete the answers to the following questions based on that live streaming. )

○ Yes

○ No (exit this questionnaire)

***B. The following items are intended to know your attitude towards streaming live marketing of agricultural products.***

Please tick **√** on the appropriate numbers according to the actual situation.

*1 represents strongly disagree; 2 represents disagree; 3 represents slightly disagree; 4 represents no comment; 5 represents slightly agree; 6 represents agree; 7 represents strongly agree.*

9.Your views on Rural Sentiment.

| I believe that the long-standing and splendid Chinese agricultural culture needs to be passed down. | 1 | 2 | 3 | 4 | 5 | 6 | 7 |
| --- | --- | --- | --- | --- | --- | --- | --- |
| Bread is the staff of life.  I believe that developing agriculture is a strategic need to ensure the food security of the country. | 1 | 2 | 3 | 4 | 5 | 6 | 7 |
| I think it is important and worthwhile to help farmers earn a good life. | 1 | 2 | 3 | 4 | 5 | 6 | 7 |
| I have a dream of idyllic life in the countryside. | 1 | 2 | 3 | 4 | 5 | 6 | 7 |

10.Your feelings when watching live streaming of agricultural products. Please complete the answers based on the question 8.

| This live streaming room shows the packaging and edible method of agricultural products in detail. | 1 | 2 | 3 | 4 | 5 | 6 | 7 |
| --- | --- | --- | --- | --- | --- | --- | --- |
| Hosts can communicate with consumers amiably and answer consumers' questions in a timely and patient manner. | 1 | 2 | 3 | 4 | 5 | 6 | 7 |
| The live streaming room has a variety of interactive activities (such as participating in the screenshot lottery and grabbing red envelopes). | 1 | 2 | 3 | 4 | 5 | 6 | 7 |
| When watching the live-stream, I can communicate the informations and contents of the agricultural product with other audiences. | 1 | 2 | 3 | 4 | 5 | 6 | 7 |
| When watching the live-stream, I was very familiar with live streaming scenes (orchards, farmland, markets, plants, etc） | 1 | 2 | 3 | 4 | 5 | 6 | 7 |
| When watching the live-stream, I felt that I was immersed in the world the hosts had created. | 1 | 2 | 3 | 4 | 5 | 6 | 7 |
| The hosts’ descriptions of the agricultural products on sale gave a strong visual impression. | 1 | 2 | 3 | 4 | 5 | 6 | 7 |
| When watching the live-stream, I felt that the products were right in front of me. | 1 | 2 | 3 | 4 | 5 | 6 | 7 |
| I believe that the informations of the agricultural products on sale were reliable. | 1 | 2 | 3 | 4 | 5 | 6 | 7 |
| I believe that the products and services provided in the live streaming room were guaranteed. | 1 | 2 | 3 | 4 | 5 | 6 | 7 |
| 3.I believe that the products I received matched the description in the live streaming room. | 1 | 2 | 3 | 4 | 5 | 6 | 7 |
| I believe that the quality of the products I purchased from this live streaming room was consistent with my expectations. | 1 | 2 | 3 | 4 | 5 | 6 | 7 |
| I believe that this live streaming room could fulfill the promises made to the consumers. | 1 | 2 | 3 | 4 | 5 | 6 | 7 |
| When listening to the hosts talk about rural life and agricultural production, I felt like these scenes, these people and things were happening around me. | 1 | 2 | 3 | 4 | 5 | 6 | 7 |
| When listening to the hosts talk about rural life and agricultural production, I felt like I was one of them. | 1 | 2 | 3 | 4 | 5 | 6 | 7 |
| I think the stories behind the agricultural production were interesting, and I was very devoted and touched. | 1 | 2 | 3 | 4 | 5 | 6 | 7 |
| When watching the live-stream, I felt like that I am talking to my old friends about the harvest. | 1 | 2 | 3 | 4 | 5 | 6 | 7 |
| 1.When watching the live-stream, I successfully placed an order as soon as possible. | 1 | 2 | 3 | 4 | 5 | 6 | 7 |
| When watching the live-stream, I added the agricultural production to my shopping cart for comparison and purchase in the future. | 1 | 2 | 3 | 4 | 5 | 6 | 7 |
| I am willing to share the live streaming link to my friends as soon as possible. | 1 | 2 | 3 | 4 | 5 | 6 | 7 |
| When the hosts or farmers mentioned the overstock of agricultural products in the live streaming room, I was willing to place an order to support the farmers. | 1 | 2 | 3 | 4 | 5 | 6 | 7 |

This is the end of the investigation. Thank you again for your support!
