# Supplementary material for: Effect of emotional factors on purchase intention in live streaming marketing of agricultural products: A moderated mediation model
Source: PLoS One. 2024 Apr 1;19(4):e0298388. doi: 10.1371/journal.pone.0298388 (PMC10984517; doi:10.1371/journal.pone.0298388)
Supplement: Attachment — Submitted filename: Response to Reviewers.docx [file pone.0298388.s005.docx]

PONE-D-23-29134

Effect of emotional factors on purchase intention in streaming live marketing of agricultural products: a moderated mediation model

PLOS ONE

Dear editor,

We sincerely thank you and all reviewers for your valuable comments that we have used to improve the quality of our manuscript.

The editor and reviewers’ comments are laid out below in italicized font and numbered sequentiallly. Our response is given in normal font and the line number where the revision is located is highlighted in blue text.

Response to editor’s comments

Comment 1. *Introduction: The necessity of research content is not reflected, and it does not indicate that the research content has not been studied by previous researchers or lacks sufficient research. The first two sections lack some logical coherence, and are suggested to be made some adjustments.*

Response: Thank you for your valuable comment! In the revision, we reviewed literature on the effects of emotional factors on purchase intention in live streaming of agricultural products(Ref. 4-10)(line85-108), and proposed that this literature is not sufficient and need to research deeply by analysing the interaction and integration effect of several emtional factors(line 109-112). These revised content could reflect the necessity of this research. At the same time, we decribed the rapid development of E-commerce live streaming in China with various data at the first paragraph of the manuscript, and the East Buy (located in the second paragraph) is a successful example of E-commerce live streaming. So the first two paragraphs become logical coherence.

Comment 2. *Literature Review: The theory used in this study, the S-O-R (Stimulus-Organism-Response) theory, lacks a certain level of innovation.*

Response: Thanks you for your valuable comment! In the revision, We introduced Consumer Engagement Theory and TAM model to increase the innovation(line 113-125). Consumer Engagement Theory explained the relationship between cognition, emotion, and/or behaviors. TAM model discussed the driving mechanism of perceived usefulness and perceived ease of use on e-commerce acceptance. Consumer Engagement Theory and TAM model, as well as SOR model, implied the effect of emotional factors on purchase intention, so conducting a research framework base on the three theories is innovative. In addtion, we moved the three theories into “Introduction” from “Literature review and Hypothese development”, because the construction of framework were based on the three theories. This movement may make the structure more reasonable and the logic clearer.

*Comment 3. Method: Based on what criteria are the data from 3-4 questions integrated into a latent variable, and there being a lack of relevant explanations and citation references for the combination of data sources.*

Response: Thanks you! It’s an important question. We used exploratory factor analysis, confirmatory factor analysis and internal consistency coefficient analysis to test internal consistency reliability, composite reliability, constructal validity(line 373), convergent validity(AVE)(line386), and discriminant validity(line 365). In the process of test, we integraed several quetions into a latent variable. In addition, we adjusted some questions according to the characteristic of agricultural products and live streaming platform, and supplemented corrosponding citation references(table 2). the contnet validity of the scale was tested through group brainstorming and expert opinion methods(line 329).

*Comment4. Conclusion and Discussion: In this section, it is suggested to reorganize the content by comparing and discussing one's own conclusions with previous research, and presenting valuable viewpoints.*

Response: This comment is very helpful! In research contribution, we compared our conclusion with Su & Wang’s research results(Ref. 50). In their study, the moderating effect of homesickness, which is the closest concept to the rural sentiment in literature, was positive. But the impact of homesickness on satisfaction is not significant. In our study, the rural sentiment has a significant positive impact on trust and resonance, demonstrating that rural sentiment has a certain substitute effect on interactivity and presence(line 599-610). Our research conclusion is a beneficial new discovery in the context of live streaming of agricultural products.

*Comment 5. Reference: Literature is scarce, and some of it is outdated. It is recommended to appropriately increase and update the references.*

Response: Thank you! It’s a valuable recommendation! In the revision, we supplemented 37 references closely related to our research content, and the total number of references become 69 (line 676-852). We have rigorously screened high-quality literature, and over 40% references are publishen in five years.

*Comment 6. Please get the paper read by a professional language editor.*

Response: Thank you for your valuable advice! We have invited a professional language editor to pulish our revision. The revision has been individually edited for grammar, punctuation, spelling, and clarity. You may verify the authenticity of this certificate on website (https://www.letpub.com/editorial-certificate) at any time using this manuscript’s unique code: PR_230724G794 v230816.

*Comment 7. Run the article on a plagiarism check software like Turnitin and eliminate similarities from other publications. The Similarity Index should be within 10 percent.*

Response: Thank you for your valuable advice! We have run this paper on Turnitin, and the Similarity Index is 7%. The originality report has been uploaded as a separate file labeled “Turnitin Originality Report”.

Response to reviewers’ comments

Comments to the Author

*1. Is the manuscript technically sound, and do the data support the conclusions?*

*The manuscript must describe a technically sound piece of scientific research with data that supports the conclusions. Experiments must have been conducted rigorously, with appropriate controls, replication, and sample sizes. The conclusions must be drawn appropriately based on the data presented.*

*Reviewer #1: No*

*Reviewer #2: Partly*

Response: Thank you for your valuable comments! In the revision, we conducted a new survey using quota sampling method, the gender, age and residence structure of the sample are consistent with the structure of Chinese netizen reported by CNNIC. The data collection work began on November 27, 2023. Due to our use of https://www.wjx.cn to collect data, it is difficult to strictly control the quota. Therefore, we calculate the composition ratio of the collected samples in real-time, and accept a deviation of no more than 5% between the sample composition ratio and the quota plan. We plan to end the survey when our total sample size reaches 1000 or more, and the total sample structure meets the quota ratio. Unitl December 26, 2023, a total of 1006 questionnaires with potential interviewees was collected. All interviewees come from a wide range of sources, covering 29 provinces and cities in Chinese Mainland except Xizang(line 333-350).

Among all the intnerviewees, 714 responders have watched live streaming of agricultural products. Since our study focus on the immediate psychological feelings of consumers watching live-stream, so the question “Have you watched agricultural product streaming live marketing more comprehensively in the past two weeks? ” was set to recogonize the appropriate research object. Then 391 responders were selected. After data review, questionnaires that did not have logical responses and were filled out randomly (with an online response time of less than 1 min) were considered invalid and further eliminated, finally 365 valid samples were ultimately obtained(line 351-364). The samples have good representativeness, and the details of sample structure was shown in Table 1.

*2. Has the statistical analysis been performed appropriately and rigorously?*

*Reviewer #1: No*

*Reviewer #2: No*

Response: Thank you for your valuable comments! We improved the survey in two ways: increase sample representativeness and improved the statistical analysis. First, we conducted the survey using quota sampling method, the gender, age and residence structure of the sample are consistent with the structure of Chinese netizen reported by CNNIC(line 333-341). The selected samples have good representativeness. Second, we used exploratory factor analysis, confirmatory factor analysis and internal consistency coefficient analysis to test internal consistency reliability, composite reliability, constructal validity, convergent validity(AVE), and discriminant validity(shown in table 4). the measurement results showed that the scales had good reliabilty and validity. Hierarchical regression was used to test the main effect(shown in table 5), sobel test and bootstrapping method was used to confirm the mediating effect(shown in table 6 and 7), and bootstrapping test was used to test the moderating effect(shown in table 8 and 9). We also do the cross-cultural analysis using heterogeneity method(shown in table 10). All these statistical analyses was performed appropriately and rigorously.

*3. Have the authors made all data underlying the findings in their manuscript fully available?*

*The PLOS Data policy requires authors to make all data underlying the findings described in their manuscript fully available without restriction, with rare exception (please refer to the Data Availability Statement in the manuscript PDF file). The data should be provided as part of the manuscript or its supporting information, or deposited to a public repository. For example, in addition to summary statistics, the data points behind means, medians and variance measures should be available. If there are restrictions on publicly sharing data—e.g. participant privacy or use of data from a third party—those must be specified.*

*Reviewer #1: Yes*

*Reviewer #2: Yes*

Response: Thank you very much for your approbation!

*4. Is the manuscript presented in an intelligible fashion and written in standard English?*

*PLOS ONE does not copyedit accepted manuscripts, so the language in submitted articles must be clear, correct, and unambiguous. Any typographical or grammatical errors should be corrected at revision, so please note any specific errors here.*

*Reviewer #1: Yes*

*Reviewer #2: Yes*

Response: Thank you very much for your approbation!

5. Review Comments to the Author

*Review #1*

*Comment 1. The contribution of the paper. The hypotheses developed in the paper is well expected. For example, H1 proposed that interaction in streaming live marketing of agricultural products has a positive impact on consumers’ purchase intention. The relationship between interaction and purchase intention has been studied by the literature. Therefore, the incremental value of the current study is not clear. I suggest the authors revise the framework.*

Response: Thank you for your valuable comments! We have revised the paper’s framework(shown in figure 1). By reviewing extensive literature on live streaming and emotional factors, we added “presence” as a independent, and “trust” as a mediator. We tested the direct effect of interactivity/presence on purchase intention, the mediating effect of trust/resonance between interactivity/presence and purchase intention, and the moderating effect of rural sentiment between interactivity/presence and trust/resonance.

Indeed, the relationship between interaction and purchase intention in live streaming has been studied by the literature (Ref. 18-20), but this paper focus on the affect mechanism of emotional factors in consumer purchase-decision process, especially the mediating role of trust and resonance. According to SOR theory, trust and resonance are Organism, while interactivity and presence are stimulus, the main effect of interactivity and purchase intention is the basis for analyzing the mediating role of trust and resonance. Therefore, we kept the analysis of the relationship between interaction and purchase intention, but paid more attention to the mediating and moderating effect of emotional factors on purchase intention.

*Comment 2. The literature review is too brief. The authors mainly discuss about stimulus–Organism–Response (SOR) in the literature review but ignored the literature on live streaming. I suggest the authors add the literature on live streaming to the paper.*

Response: Thank you! the comment is very helpful! We added the literature on live streaming as well as corresponding theory besides SOR model. First, we added literature on the effects of emotional factors on purchase intention in live streaming of agricultural products (line 85-108). We have rigorously screened high-quality literature, and excluded outdataed literature. Second, we added Consumer Engagement Theory and TAM model to strengthen the theoretical foundation of this paper(line 113-125). Base on the review of Consumer Engagement Theory and TAM model, as well as SOR model, the literature on live streaming became sufficient to some extent. It should be noticed that we moved the literature review and three theories into “Introduction” from “literature review”, because the construction of framework and selection of emotional factors were based on the literature review and three theories. This movement may make the structure more reasonable and the logic clearer.

*Comment 3. The theoretical support for the survey questions. The literature to support the development of the survey questions seems not very solid. I suggest the authors develop the survey questions using high-cited articles.*

Response: Thank you for your valuable recommendation! Before develping the survery questions, we add two variables, i. e “presence” and “trust”, according to reviewer #2’ comments. Then, we reviewed a large amount of literature on variables in this article, including “purchase intention”, “interactivity”, “presence”, “trust”, “resonance”, and “rural sentiment”, and explored multiple measurement items that can reflect these variables. Based on these measurement items, we proposed creative measurement items for each variable by combining with the context of live streaming of agrecultral product. This literature we refered to is of high quality and highly cited, ensuring the solidity of the measurement items. Variable measurement and source were shown in table 2.

Review #2

*Consider enlarging the sample size and diversifying demographic representation to enhance the external validity of findings, providing more nuanced insights into how emotional factors influence purchase intention across various consumer groups. Explore the feasibility of conducting a cross-cultural analysis, recognizing that different cultures may respond differently to emotional cues in marketing. This could offer valuable insights into the universality or cultural specificity of emotional factors in agricultural product marketing. Assess the feasibility of a longitudinal study to capture changes in consumer behavior over time, providing insights into the long-term impact of emotional factors on purchase decisions amid evolving market trends and technological advancements. Incorporate qualitative methods, such as in-depth interviews or focus group discussions, to gain a deeper understanding of consumers' emotional responses. Qualitative data can complement quantitative findings, offering richer insights into the nuances of emotional experiences during live streaming. Expand the scope of emotional factors studied beyond interaction, resonance, and rural sentiment. Consider exploring additional emotional factors like trust, excitement, or nostalgia that may play a role in agricultural product marketing, providing a more comprehensive Please discuss the potential policy implications arising from the findings. For example, if emotional factors significantly impact agricultural product sales, policymakers may consider supporting initiatives that enhance emotional engagement in the agricultural sector. Provide practical recommendations for marketers based on the study's findings. Offer insights into how agricultural product marketers can leverage emotional factors in their live streaming strategies, presenting actionable recommendations that industry professionals can apply.*

Response: Thanks you! We gain a lot from your valuable and helpful comments! In order to respond to your comments organized and clearly, we divied them into six items and responded them separately.

**Item 1**. *Consider enlarging the sample size and diversifying demographic representation to enhance the external validity of findings, providing more nuanced insights into how emotional factors influence purchase intention across various consumer groups.*

Response: This is a vital issue for this study! In order to diversity demographic representation of this surey, we conducted a new survey using quota sampling method. The gender, age and residence structure of the sample are consistent with the structure of Chinese netizen reported by CNNIC. The data collection work began on November 27, 2023. Due to our use of https://www.wjx.cn to collect data, it is difficult to strictly control the quota. Therefore, we calculate the composition ratio of the collected samples in real-time, and accept a deviation of no more than 5% between the sample composition ratio and the quota plan. We plan to end the survey when our total sample size reaches 1000 or more, and the total sample structure meets the quota ratio. Unitl December 26, 2023, a total of 1006 questionnaires with potential interviewees was collected. All interviewees come from a wide range of sources, covering 29 provinces and cities in Chinese Mainland except Xizang(line 333-350).

Among all the intnerviewees, 714 responders have watched live streaming of agricultural products. Since our study focus on the immediate psychological feelings of consumers watching live-stream, so the question “Have you watched agricultural product streaming live marketing more comprehensively in the past two weeks? ” was set to recogonize the appropriate research object. Then 391 responders were selected. After data review, questionnaires that did not have logical responses and were filled out randomly (with an online response time of less than 1 min) were considered invalid and further eliminated, finally 365 valid samples were ultimately obtained. The samples have good representativeness, and ensure the external validity of the study(line 351-364). The details of sample structure was shown in Table 1.

The measurement results show that, the scale has good internal consistency reliability, composite reliability, constructal validity, which could provide more nuanced insights into how emotional factors influence purchase intention across various consumer groups.

**Item 2**. *Explore the feasibility of conducting a cross-cultural analysis, recognizing that different cultures may respond differently to emotional cues in marketing. This could offer valuable insights into the universality or cultural specificity of emotional factors in agricultural product marketing.*

Response: Thank you for your valuable advice! In the light of your suggestion, we conducted a cross-cultural analysis. Considering that different cultural backgrounds may lead to differences in consumers’ emotional responses when watching live streaming of agricultural products, we explored the feasibility of conducting a cross-cultural analysis. People who lived in countryside may have intenser emotions about farming culture and agricultural products. So, we divided the respondents into groups of urban and rural to conduct a comparison through regression analysis. The result presents the coefficient from rural sentiment to trust in the rural group is significant(line 580-584). There are seven remarkable differences between urban and rural groups, which could provide valuable insights into the universality or cultural specificity of emotional factors in agricultural product marketing.

**Iterm 3.** *Assess the feasibility of a longitudinal study to capture changes in consumer behavior over time, providing insights into the long-term impact of emotional factors on purchase decisions amid evolving market trends and technological advancements.*

Response: Thanks for your helpful comment. This paper had examined the immediate emotional response of consumers in live streaming, the follow-up tracking of consumer behavior doesn’t carry out. Longitudinal study might obtain robust findings, but for anonymous online surveys, we are unable to obtain the contact information of the respondents and cannot conduct secondary visits. Therefore, the study of time series cannot be achieved in this study. Therefore, this subsequent study will consider capturing changes in consumer behavior over time. For example, we plan to perform regular interviews with consumers who place orders in live-streaming room that uses emotional factors to influence purchase intentions,so that further gaining an deep understanding of how emotional factors influence purchase decisions amid evolving market trends and technological advancement.

***Iterm 4.*** *Incorporate qualitative methods, such as in-depth interviews or focus group discussions, to gain a deeper understanding of consumers' emotional responses. Qualitative data can complement quantitative findings, offering richer insights into the nuances of emotional experiences during live streaming.*

Response: Thanks for your helpful comment. This paper mainly obtained samples through e-questionnaires and conducted the quantitative research. The questionnaire designed in this paper were mainly referred to mature scales from existing literature, and expert opinions were consulted during revision. The content validity of the questionnaire was good. We will add qualitative methods to our future study, such as in-depth interviews and focus group discussions. In addition, we will use the real-time comments of the audience in the live streaming room to gain an in-depth understanding of consumers’ emotional response, so as to make a preliminary judgement on whether the emotional factors will affect the purchase intention.

***Iterm 5.*** *Expand the scope of emotional factors studied beyond interaction, resonance, and rural sentiment. Consider exploring additional emotional factors like trust, excitement, or nostalgia that may play a role in agricultural product marketing, providing a more comprehensive.*

Response: Thank you for your valuable comment! In the light of your suggestion, we reviewed extensive literature about live streaming of agricultral product, and added two emotional factors into our study, that is, “presence” as an independent variable and “trust” as a mediating variable (shown in figure 1). We reviewed the literature on the effect mechanism of emotional factors on purchase intention, and found “presence” is a essential variable to enhance consumers’ purchase intention (Ref. 4), and presence could effect purchase intention through trust and rensonance( Ref. 23, 24, 25, 34, 44). We also found the mediating role of “trust” between interactiviety/presence and purchase intention(Ref. 8, 23, 24, 28, 31). The effect of presence and trust were also mentioned in Consumer Engagement Theory and TAM model, and SOR model(Ref. 11-15). Therefore, we believe the introduction of “presence” and “trust” is reasonable, and play more comprehensive role in agricultural product marketing research.

***Iterm 6.*** *Please discuss the potential policy implications arising from the findings. For example, if emotional factors significantly impact agricultural product sales, policymakers may consider supporting initiatives that enhance emotional engagement in the agricultural sector. Provide practical recommendations for marketers based on the study's findings. Offer insights into how agricultural product marketers can leverage emotional factors in their live streaming strategies, presenting actionable recommendations that industry professionals can apply.*

Response: Thank you very much. This paper has supplemented the potential policy implications according to the findings. For example, government officials who act as “specially invited guest and assistant” in the live streaming room will make consumers trust the quality of agricultural products(line 646-650). And they should also propagate the regional history, culture, and social customs, which will greatly increase the attractiveness of the region and products(line 651-652 ).

And we provide agricultural product marketers with practical recommendations about how to leverage emotional factors properly in their live streaming strategies. The hosts might chat with consumers friendly, provide credible information and encourage bullet screen and sharing thoughts(line633-635) to enhance the presence, trust and resonance of consumers. they might describe the rural life, personal rural experience, and stories and history to arouse consumers’ rural sentiment and resonance(line 636-639). The scene of the live-stream room must be designed and set up carefullyso as to improve consumers’ presence(line 630-632).

That is all response to the editor and reviewers comments.

We look forward to hearing from you regarding our submission. We would be glad to respond to any further questions and commers that you may have.

Best regards,

Your sincerely

Weibao Li
